# High Erythropoiesis Resistance Index Is a Significant Predictor of Cardiovascular and All-Cause Mortality in Chinese Maintenance Hemodialysis Patients

**DOI:** 10.1155/2020/1027230

**Published:** 2020-11-26

**Authors:** Xiangxue Lu, Jialing Zhang, Shixiang Wang, Qian Yu, Han Li

**Affiliations:** Department of Blood Purification, Beijing Chao-Yang Hospital, Capital Medical University, Beijing 100020, China

## Abstract

**Background:**

Renal anemia is a common complication of hemodialysis patients. Erythropoietin (EPO) hyporesponsiveness has been recognized as an important factor to poor efficacy of recombinant human erythropoietin in the treatment of renal anemia. More importantly, increased erythropoiesis resistance index (ERI) may be associated with inflammation and increased mortality.

**Objective:**

The objective of this research was to investigate correlated factors of EPO responsiveness and to clarify the relationships between EPO hyporesponsiveness and cardiovascular mortality and all-cause mortality among maintenance hemodialysis patients.

**Methods:**

This prospective cohort study enrolled 276 maintenance hemodialysis patients for a 55-month follow-up to investigate the factors related to ERI and its relationship to all-cause mortality and cardiovascular mortality.

**Results:**

ERI was positively correlated with predialysis serum high-sensitivity C-reactive protein (*r* = 0.234, *p* < 0.001), alkaline phosphatase (*r* = 0.134, *p* = 0.028), and ferritin (*r* = 0.155, *p* = 0.010) and negatively correlated with albumin (*r* = −0.206, *p* < 0.001) and creatinine (*r* = −0.232, *p* < 0.001). As multiple linear regression showed, predialysis serum albumin, high-sensitivity C-reactive protein, ferritin, and creatinine were independent correlated factors of ERI (*p* < 0.05). Kaplan–Meier curves showed that the cumulative incidences of both cardiovascular mortality and all-cause mortality were significantly higher in patients with ERI > 11.04 IU/kg/w/g/dL (both *p* < 0.01). The high ERI group was significantly associated with higher risk for all-cause mortality (OR 1.781, 95% CI 1.091 to 2.910, *p* = 0.021) and cardiovascular mortality (OR 1.972, 95% CI 1.139 to 3.417, *p* = 0.015) after adjusting for confounders.

**Conclusions:**

Predialysis serum albumin, high-sensitivity C-reactive protein, ferritin, and creatinine were independent correlated factors of EPO responsiveness among maintenance hemodialysis patients. Patients with higher ERI values had a higher all-cause mortality rate and cardiovascular mortality rate.

## 1. Introduction

Renal anemia is a common complication of chronic kidney disease (CKD). The incidence and severity of anemia increase with the decline of renal function, and more than 90% of patients with end-stage renal disease have been diagnosed with anemia [1]. It not only affects the quality of life but also increases cardiovascular events and all-cause mortality [2, 3]. The main causes of anemia in maintenance hemodialysis (MHD) patients are erythropoietin and iron deficiency [4].

The application of erythropoiesis-stimulating agents (ESAs) such as recombinant human erythropoietin (rHuEPO) has dramatically improved the management of anemia in MHD patients. But a proportion of patients do not respond well to erythropoietin, which is called erythropoietin (EPO) hyporesponsiveness or EPO resistance [5]. EPO hyporesponsiveness/resistance is a term to describe the failure to reach the targeted hemoglobin level despite higher than usual doses of EPO or a continuous need for higher EPO doses to maintain achieved hemoglobin level. [6] Erythropoiesis resistance index (ERI) is an important evaluation index to evaluate the EPO responsiveness which is calculated by the dose of EPO and the level of hemoglobin.

Studies have shown that many factors may be associated with EPO responsiveness such as serum albumin level, inflammatory response, secondary hyperparathyroidism, and iron deficiency [7]. In a metaregression analysis, higher doses of EPO were found to be associated with increased rate of hypertension, stroke, and thrombotic events in CKD patients [8]. Chung et al. [9] demonstrated that in MHD patients, EPO resistance was associated with left ventricular mass index, left ventricular systolic function, and cardiovascular (CV) events. It was also found that EPO hyporesponsiveness could be a predictor of all-cause mortality in MHD patients [10].

But it is still not clear whether there are other factors associated with EPO hyporesponsiveness, and the association between all-cause mortality and cardiovascular mortality should be further confirmed. Therefore, we performed this study to examine the factors associated with EPO hyporesponsiveness in MHD patients and to clarify the relationships between EPO hyporesponsiveness and cardiovascular mortality and all-cause mortality in MHD patients.

## 2. Subjects and Methods

### 2.1. Patients and Study Design

A total of 276 maintenance hemodialysis patients in December 2015 in the Department of Blood Purification, Beijing Chao-Yang Hospital, Capital Medical University, were included in this prospective cohort study. The inclusion criteria were as follows: (1) age > 18 years, (2) hemodialysis treatment duration > 3 months and in stable condition, (3) use of arteriovenous fistula, and (4) anuria. The exclusion criteria were as follows: (1) severe cardiovascular or cerebrovascular diseases, (2) infectious diseases within one month, (3) active liver diseases or cancer, (4) recent blood transfusion or surgical procedures, (5) active hemorrhage or pure red cell aplastic, (6) no treatment with rHuEPO, and (7) limb deficiency.

All patients received hemodialysis 3 times per week for 4 hours each time, using sugar-free bicarbonate dialysates and heparin anticoagulants during hemodialysis. The dialysate flow was 500 mL/min, and the blood flow was 200~350 mL/min. The dialysate ingredients were as follows: sodium 140 mmol/L, potassium 2.0 mmol/L, calcium 1.25 mmol/L, and magnesium 0.5 mmol/L. All patients had Kt/V > 1.2.

The study adhered to the Declaration of Helsinki and was approved by the Ethics Committee of Beijing Chao-Yang Hospital, Capital Medical University. The written informed consents were obtained from all participants.

### 2.2. Data Collection

The patients' demographic data including age, gender, weight, duration of dialysis, the cause of end-stage renal disease, and treatment dose of rHuEPO were recorded. All laboratory indicators including predialysis serum albumin, prealbumin, creatinine, blood urea nitrogen (BUN), alkaline phosphatase (ALP), phosphorus, total calcium, high-sensitivity C-reactive protein (hs-CRP), intact parathyroid hormone (iPTH), ferritin, serum iron, total iron binding capacity, folic acid, and blood routine tests were detected before hemodialysis. The predialysis blood samples were drawn from the venous blood line in patients with arteriovenous fistula after an overnight fast of at least 12 hours.

The corrected serum calcium level was measured as follows: corrected serum calcium level (mg/dL) = measured total calcium level (mg/dL) − serum albumin level (g/dL) + 4 mg/dL. [11] The transferrin saturation was measured as follows: transferrin saturation (%) = predialysis serum iron (*μ*mol/L)/total serum iron binding capacity (*μ*mol/L) × 100%. The EPO responsiveness was evaluated by ERI. ERI = total rHuEPO dose per week (IU)/body weight (kg)/hemoglobin (g/dL) [12]. All patients were divided into 2 groups by the median value of the ERI.

### 2.3. Outcome Data Collection

The outcomes of this study were defined as all-cause death and cardiovascular death. The subjects were followed up until May 31, 2020, for 55 months. CV death was defined as death due to ischemic heart disease, arrhythmias, sudden cardiac death, congestive heart failure, other heart disease, or cerebrovascular events.

### 2.4. Statistical Analysis

The SPSS 23.0 statistics package for Windows was used for statistical analysis. Normally distributed continuous variables were expressed as the mean ± SD, and Student *t* tests were used for comparing the mean values of the data. Nonnormally distributed variables were expressed as median (interquartile range), and the differences between the two groups were analyzed by the Mann–Whitney *U* test. Categorical variables were expressed in terms of frequency and percentage, and the chi-square test was used. Pearson or Spearman correlation was used to analyze the correlation between ERI and other clinical indicators. Multiple linear regression analysis was used for independent correlation factors of ERI. The Kaplan–Meier method was used with the log-rank test to evaluate the association between ERI and death. The Cox proportional hazards regression model was used to calculate an odds ratio (OR) with a 95% confidence interval (CI) for mortality risk, using the low ERI group as the reference value. *p* < 0.05 was considered statistically significant.

## 3. Results

### 3.1. Characteristics of the Patients

A total of 276 MHD patients were enrolled (age 58.37 ± 13.91 years), including 150 males (54.3%) and 126 females (45.7%). Duration of dialysis was 95.50 (86.75) months, and ERI was 12.57 ± 6.13 IU/kg/w/g/dL. The causes of end-stage renal disease were chronic glomerulonephritis in 118 patients (42.8%), diabetic nephropathy in 52 patients (18.8%), hypertensive nephrosclerosis in 37 patients (13.4%), chronic interstitial nephritis in 21 patients (7.6%), and other renal diseases in 48 patients (17.4%).

### 3.2. Characteristics of Patients in the Low ERI Group and the High ERI Group

The median ERI value was 11.04 IU/kg/w/g/dL. All patients were divided into the low ERI group (ERI ≤ 11.04 IU/kg/w/g/dL) and the high ERI group (ERI > 11.04 IU/kg/w/g/dL) according to the median ERI value. As Table 1 revealed, age, predialysis serum hs-CRP, ALP, and ferritin in the patients of the high ERI group were higher than those of the low ERI group, while Alb, prealbumin, and creatinine were lower in the high ERI group compared to the low ERI group (*p* < 0.05). However, there was no significant difference between the two groups in duration of dialysis, TSAT, predialysis serum BUN, and predialysis serum folic acid (*p* > 0.05).

### 3.3. Correlated Factors of ERI in MHD Patients

Pearson and Spearman correlation analyses were performed to assess the relationships between ERI and other factors. As shown in Table 2, ERI was positively correlated with predialysis serum high-sensitivity C-reactive protein (*r* = 0.234, *p* < 0.001), alkaline phosphatase (*r* = 0.134, *p* = 0.028), and ferritin (*r* = 0.155, *p* = 0.010) and negatively correlated with albumin (*r* = −0.206, *p* < 0.001) and creatinine (*r* = −0.232, *p* < 0.001). But ERI had no correlation with age or prealbumin (*p* > 0.05).

In multiple linear regression model, predialysis serum Alb, hs-CRP, ferritin, and creatinine were independent correlated factors of ERI as shown in Table 3 (*p* < 0.05).

### 3.4. Association of ERI with All-Cause and Cardiovascular Death in MHD Patients

**Figure 1:**
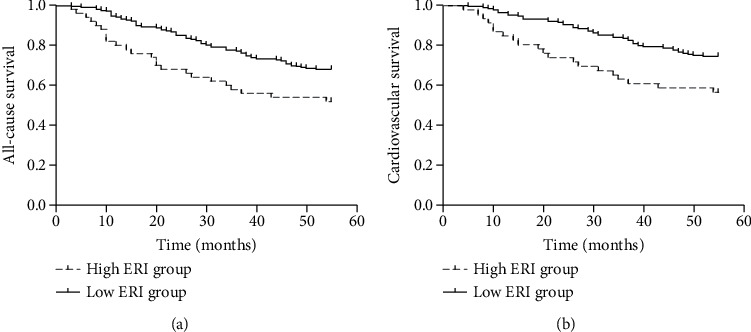
Kaplan–Meier plots for all-cause mortality and cardiovascular mortality in MHD patients. (a) Patients with ERI > 11.04 had significantly higher all-cause mortality (log rank = 6.719; *p* < 0.01). (b) Patients with ERI > 11.04 had significantly higher cardiovascular mortality (log rank = 7.800; *p* < 0.01).

By the end of the follow-up, 90 of the 276 patients died, including 68 cardiovascular deaths, 4 gastrointestinal bleeding deaths, 6 tumor deaths, and 12 deaths from other causes. When patients were divided into 2 groups according to the median ERI value (below or above the median value of 11.04), the Kaplan–Meier curves indicated that patients with higher ERI values had a higher all-cause mortality rate (log rank = 6.719; *p* < 0.01) and a higher cardiovascular mortality rate (log rank = 7.800; *p* < 0.01) (Figures 1(a) and 1(b)). Univariate and multivariate Cox regression analyses for mortality risk in MHD patients are shown in Table 4. In multivariate Cox regression analysis, the adjusted OR for all-cause mortality in the high ERI group was 1.781 (95% CI 1.091 to 2.910, *p* = 0.021), even after adjusting for confounders while the OR was 1.972 (95% CI 1.139 to 3.417, *p* = 0.015) for cardiovascular mortality.

## 4. Discussion

The results of this study demonstrated the relationship between comorbidity factors and erythropoietin resistance in MHD patients and its negative effect on survival. ERI was used to evaluate ESA hyporesponsiveness and regarded as a prognostic factor for mortality. Correlation analysis found that ERI had a statistically positive correlation with hs-CRP, ferritin, and ALP and a negative correlation with ALB and creatinine. We further found that elevated ERI was a significant predictor of all-cause mortality and cardiovascular mortality in MHD patients.

The pathogenesis of renal anemia is complex, and absolute or relative deficiency of endogenous EPO is the main cause of renal anemia. Renal anemia not only leads to fatigue, palpitation, and dyspnea and affects the quality of life among hemodialysis patients but is also an important sign of poor prognosis in hemodialysis patients [13, 14]. Clinically, some patients with renal anemia have poor improvement in hemoglobin level after treatment with weight-dependent rHuEPO, or the dose of rHuEPO per body weight required to maintain normal hemoglobin levels is significantly higher than that in other patients with renal anemia, which is a phenomenon known as EPO hyporesponsiveness/resistance [15]. It has been suggested that the deficiency of hemoglobin synthesis materials caused by absolute or functional iron deficiency is an important reason for the poor effect of rHuEPO in the treatment of renal anemia [16]. Meanwhile, anti-rHuEPO antibodies were found in some patients, leading to rHuEPO-associated pure red blood cell aplastic anemia, which is also the cause of EPO hyporesponsiveness [17].

In our study, hs-CRP, a marker of inflammation, was significantly associated with ERI in both correlation analysis and multiple linear regression analysis. A previous study including continuous ambulant peritoneal dialysis (CAPD) patients also reported that CRP was the most important predictor of EPO hyporesponsiveness [18]. A low-grade inflammation was common in dialysis patients, which was due to the presence of oxidative stress, uremia toxin deposition, metabolic disorder, and other pathological states, leading to the disorder of cytokines [19]. Erythropoiesis might be blocked by inflammatory cytokines, such as interleukin-1 (IL-1), interleukin-6 (IL-6), and tumor necrosis factor-alpha (TNF-*α*). Elevated IL-6 and TNF-*α* levels might result in disorder of iron metabolism and iron stores in the reticuloendothelial system. Such cytokines might also damage red blood cells directly and induce apoptosis in marrow. It is important to prevent dialysates from bacterial contamination for dialysis patients. And more inflammatory mediators should be considered to jointly increase the risk of ESA resistance.

Similar to previous studies [20–22], we found that serum ferritin, a marker of iron stores, was an important cause of resistance to EPO treatment and a predictor of all-cause mortality in dialysis patients. However, the association between high ferritin and mortality was attenuated after adjustment for markers of inflammation. It was shown that ferritin might interact with inflammation to result in ESAs hyporesponsiveness by activating the NF-*κ*B pathway [23]. However, the relationship between ferritin and ERI was still on debate while ferritin light chain might protect against endotoxemia [24]. More attention should be paid to this field.

We also found that ERI had a negative correlation with predialysis serum albumin and creatinine in our study. Albumin and creatinine were both indicators of nutrition status which was related to the development of malnutrition-inflammation-atherosclerosis (MIA) syndrome. Hypoalbuminemia was a relative predictor of ESA responsiveness and survival in hemodialysis patients [25–27], which was probably also associated with inflammatory reaction. Predialysis serum creatinine level, a potential surrogate of muscle mass, was negatively related to mortality in HD and peritoneal dialysis patients [28, 29]. Our findings indicated that nutrition supplement might be useful to improve ESA resistance.

Serum ALP was generally regarded as an indicator of renal bone disease in CKD patients accompanying hyperparathyroidism [30]. Hyperparathyroidism might affect EPO synthesis, erythrocyte survival, and bone marrow fibrosis contributing to ESA resistance [31]. However, ALP was not found to be an independent risk factor for ERI in multiple linear regression analysis in this study. Serum ALP is a relative correlated factor for serum iPTH level. But serum iPTH level was not significantly different between groups. The mean values of iPTH in the two groups were both within the targeted range recommended by Kidney Disease: Improving Global Outcomes (KDIGO) Clinical Practice Guideline [32]. So the comorbidities and nutrition status should be taken into consideration. And it probably indicated the effectiveness of therapy with calcimimetics or parathyroidectomy.

Moreover, the current study demonstrated that the risk for all-cause and cardiovascular mortality was significantly different between groups. ESA resistance could be a risk factor for all-cause and cardiovascular mortality after adjusting for confounders. In the previous studies, the relationship between mortality and ERI was controversial. Some studies have shown that ESA resistance was associated with increased mortality [33, 34]. A meta-analysis containing 31 trials also reported that ESA dose might be positively associated with all-cause mortality independent of hemoglobin level. [8] However, another study including 320 HD patients suggested that low Hb levels and high-dose epoetin doses were significantly associated with mortality while ERI was not [35]. ESA doses are variable among anemic patients. Erythropoietin receptors have been found not only on human endothelial cells but also on tumor cells. Higher concentration of ESA might increase the risk for mortality. It is essential to determine an appropriate method to estimate ESA responsiveness for renal anemia management. Considering ERI was calculated based on baseline data, it might be influenced by the long-term follow-up period and inflammatory and nutritional status. According to our findings, ERI might provide significant information to help predict cardiovascular disease and all-cause mortality in MHD patients.

However, this was a nonrandomized, single-center study that included a relatively small number of patients, which is the limitation of the study.

In conclusion, ERI was positively associated with hs-CRP and ferritin, while serum ALB and creatinine protect from ESA resistance in HD patients. Furthermore, high ERI was a strong predictor for all-cause mortality and cardiovascular mortality in MHD patients.

## Figures and Tables

**Table 1:** Characteristics of patients in the low ERI group and the high ERI group.

Variables	Low ERI group (*n* = 138)	High ERI group (*n* = 138)	Statistics	*p* value
Age (years)	56.57 ± 13.14	60.17 ± 14.46	-2.165	0.031^∗^
Sex (male)	72 (52.17%)	78 (56.52%)	0.526	0.468
Duration of dialysis (months)	94.50 (80.75)	96.00 (90.50)	-0.221	0.825
WBC (×10^9^)	6.47 ± 1.94	6.09 ± 1.77	1.668	0.093
NEUT (%)	66.36 ± 8.27	67.01 ± 8.91	-0.604	0.546
Alb (g/L)	39.69 ± 3.35	37.49 ± 3.72	5.612	<0.001^∗^
Prealbumin (g/L)	0.33 ± 0.08	0.31 ± 0.09	2.621	0.009^∗^
BUN (mmol/L)	23.60 ± 4.71	23.28 ± 5.80	0.498	0.619
hs-CRP (mmol/L)	2.63 (4.91)	4.49 (6.69)	-3.061	0.002^∗^
iPTH (pg/mL)	268.00 (197.30)	290.65 (348.58)	-1.186	0.236
ALP (U/L)	104.00 (45.50)	111.00 (43.00)	-2.249	0.024^∗^
P (mmol/L)	1.88 ± 0.53	1.91 ± 0.58	-0.544	0.587
cCa (mg/dL)	9.17 ± 1.07	9.01 ± 0.97	1.302	0.194
cCa × P (mg^2^/dL^2^)	58.54 ± 18.06	56.1 ± 17.02	1.150	0.251
sFerr (*μ*g/L)	371.61 ± 112.30	402.43 ± 119.58	-2.207	0.028^∗^
TSAT (%)	31.99 ± 10.65	33.02 ± 10.57	-0.659	0.511
Folic acid (nmol/L)	24.50 (17.32)	24.50 (17.80)	-1.037	0.300
Creatinine (*μ*mol/L)	937.45 ± 198.59	875.75 ± 191.84	2.625	0.009^∗^
ERI (IU/kg/w/g/dL)	8.37 (4.19)	16.615 (6.93)	-14.364	<0.001^∗^

^∗^Significant difference between the low ERI group and the high ERI group, *p* < 0.05. WBC: white blood count; NEUT: neutrophil; Alb: predialysis serum albumin; prealbumin: predialysis serum prealbumin; BUN: predialysis blood urea nitrogen; hs-CRP: predialysis serum high-sensitivity C-reactive protein; iPTH: predialysis serum intact parathyroid hormone; ALP: predialysis serum alkaline phosphatase; P: predialysis serum phosphorus; cCa: predialysis corrected serum calcium; sFerr: predialysis serum ferritin; TSAT: predialysis transferrin saturation; folic acid: predialysis serum folic acid; creatinine: predialysis serum creatinine.

**Table 2:** Correlation analysis for variables and ERI in MHD patients.

Variables	*r* value	*p* value
Age (years)	0.112	0.064
Alb (g/L)	-0.206	<0.001^∗^
hs-CRP (mmol/L)	0.234	<0.001^∗^
Prealbumin (g/L)	-0.111	0.066
ALP (U/L)	0.134	0.028^∗^
SFerr (*μ*g/L)	0.155	0.010^∗^
Creatinine (*μ*mol/L)	-0.232	<0.001^∗^

^∗^The correlation was statistically significant, *p* < 0.05. Alb: predialysis serum albumin; prealbumin: predialysis serum prealbumin; hs-CRP: predialysis serum high-sensitivity C-reactive protein; ALP: predialysis serum alkaline phosphatase; sFerr: predialysis serum ferritin; creatinine: predialysis serum creatinine.

**Table 3:** Multiple linear regression analysis for ERI.

	Unstandardized coefficient	Standard error	Standardized coefficient	*t* value	95% CI	*p* value	VIF value
Alb (g/L)	-0.276	0.095	-0.166	-2.907	-0.463~-0.089	0.004^∗^	1.055
hs-CRP (mmol/L)	0.411	0.090	0.260	4.583	0.234~0.588	<0.001^∗^	1.035
ALP (U/L)	0.002	0.006	0.019	0.343	-0.010~0.014	0.732	1.016
sFerr (*μ*g/L)	0.006	0.003	0.119	2.095	0.000~0.012	0.037^∗^	1.043
Creatinine (*μ*mol/L)	-0.006	0.002	-0.185	-3.253	-0.009~-0.002	0.001^∗^	1.043
Constant	23.863	3.920		6.087	16.144~41.582	<0.001^∗^	

^∗^The regression coefficient was statistically significant, *p* < 0.05. Adjusted for serum albumin, hs-CRP, ALP, sFerr, and creatinine. Alb: predialysis serum albumin; hs-CRP: predialysis serum high-sensitivity C-reactive protein; ALP: predialysis serum alkaline phosphatase; sFerr: predialysis serum ferritin; creatinine: predialysis serum creatinine.

**Table 4:** Cox proportional HR for all-cause mortality and cardiovascular mortality.

	All-cause mortality^a^				Cardiovascular mortality^b^			
	Crude OR (95% CI)	*p*	Adjusted OR (95% CI)	*p*	Crude OR (95% CI)	*p*	Adjusted OR (95% CI)	*p*
ERI ≤ 11.04	Reference				Reference			
ERI > 11.04	1.895 (1.188-3.025)	0.007	1.781 (1.091-2.910)	0.021	2.188 (1.298-3.687)	0.003	1.972 (1.139-3.417)	0.015

^a^Adjusted for predialysis serum albumin, high-sensitivity C-reaction protein, magnesium, predialysis serum ferritin, serum transferrin saturation, predialysis corrected calcium, phosphorus, high-density lipoprotein, low-density lipoprotein, total cholesterol, and triglyceride. ^b^Adjusted for predialysis serum albumin, predialysis serum ferritin, serum transferrin saturation, predialysis corrected calcium, phosphorus, high-density lipoprotein, low-density lipoprotein, total cholesterol, and triglyceride. ERI: erythropoiesis resistance index; OR: odds ratios; CI: confidence interval; *p*: *p* value.

## Data Availability

Data was available on request.
